# Three-Dimensional Postoperative Results Prediction for Orthognathic Surgery through Deep Learning-Based Alignment Network

**DOI:** 10.3390/jpm12060998

**Published:** 2022-06-18

**Authors:** Seung Hyun Jeong, Min Woo Woo, Dong Sun Shin, Han Gyeol Yeom, Hun Jun Lim, Bong Chul Kim, Jong Pil Yun

**Affiliations:** 1 Advanced Mechatronics R&D Group, Korea Institute of Industrial Technology (KITECH), Gyeongsan 38408, Korea; shjeong@kitech.re.kr (S.H.J.); wmw@kitech.re.kr (M.W.W.); 2 School of Computer Science and Engineering, Kyungpook National University, Daegu 41566, Korea; 3 Department of Oral and Maxillofacial Surgery, Daejeon Dental Hospital, College of Dentistry, Wonkwang University, Daejeon 35233, Korea; sdssoft@gmail.com (D.S.S.); hun216@wku.ac.kr (H.J.L.); 4 Department of Oral and Maxillofacial Radiology, Daejeon Dental Hospital, College of Dentistry, Wonkwang University, Daejeon 35233, Korea; hangyeol1214@gmail.com; 5 KITECH School, University of Science and Technology, Daejeon 34113, Korea

**Keywords:** deep learning, dentofacial deformities, orthognathic surgery, CT X-ray

## Abstract

To date, for the diagnosis of dentofacial dysmorphosis, we have relied almost entirely on reference points, planes, and angles. This is time consuming, and it is also greatly influenced by the skill level of the practitioner. To solve this problem, we wanted to know if deep neural networks could predict postoperative results of orthognathic surgery without relying on reference points, planes, and angles. We use three-dimensional point cloud data of the skull of 269 patients. The proposed method has two main stages for prediction. In step 1, the skull is divided into six parts through the segmentation network. In step 2, three-dimensional transformation parameters are predicted through the alignment network. The ground truth values of transformation parameters are calculated through the iterative closest points (ICP), which align the preoperative part of skull to the corresponding postoperative part of skull. We compare pointnet, pointnet++ and pointconv for the feature extractor of the alignment network. Moreover, we design a new loss function, which considers the distance error of transformed points for a better accuracy. The accuracy, mean intersection over union (mIoU), and dice coefficient (DC) of the first segmentation network, which divides the upper and lower part of skull, are 0.9998, 0.9994, and 0.9998, respectively. For the second segmentation network, which divides the lower part of skull into 5 parts, they were 0.9949, 0.9900, 0.9949, respectively. The mean absolute error of transverse, anterior–posterior, and vertical distance of part 2 (maxilla) are 0.765 mm, 1.455 mm, and 1.392 mm, respectively. For part 3 (mandible), they were 1.069 mm, 1.831 mm, and 1.375 mm, respectively, and for part 4 (chin), they were 1.913 mm, 2.340 mm, and 1.257 mm, respectively. From this study, postoperative results can now be easily predicted by simply entering the point cloud data of computed tomography.

## 1. Introduction

Dentofacial dysmorphosis is characterized by retrognathism, prognathism, and asymmetry [1,2,3]. Various orthognathic surgery techniques have been applied for treatment [4,5]. An accurate and stereoscopic diagnosis of the patient’s current condition is required prior to operation [6]. Until now, orthognathic surgery has mainly depended on linear and angular measurements for the diagnosis and planning of therapeutic procedures [6,7,8]. These measurements rely on the identification of several landmarks on cephalometric images, which are then applied to define the measurements. This is not only complicated and cumbersome, but it also takes a long time because it is greatly affected by proficiency. 

Deep learning has developed rapidly in recent years, making it possible to automatically extract information in the medical field from diagnoses using medical imaging and pattern analysis [9,10,11,12,13,14,15]. Deep neural networks (DNNs), a type of deep learning, have been widely applied to medical images because of their high performance in detection, classification, and segmentation [16,17,18,19,20]. It can reduce the labor of experts while detecting image information that may be missed by humans. Deep learning research related to the diagnosis and planning of dentofacial dysmorphosis is being actively conducted [21,22,23]. However, most have focused only on finding reference points, planes, and angles using conventional methods [21,22,23]. This may have been due to their failure to grasp the true nature of DNNs. Hence, we propose a method that better fits DNN characteristics. In this study, the authors introduced the prediction of postoperative results for orthognathic surgery that are not bound by points, planes, and angles.

The present method is used to predict postoperative orthognathic surgery using a DNN trained with actual three-dimensional (3D) point cloud data. In contrast to a previous study using artificially generated 3D point cloud data [24], the present method considers actual pre- and postoperative surgery data for training. In doing so, the present method resolved three main issues. 

The first issue is related to the generation of training data from actual pre- and postoperative surgery data. In this study, we attempted to learn from actual data, not from simulation data that arbitrarily caused skull deformations in previous studies [24]. However, there is a problem when trying to generate training data from actual data: the point cloud of pre- and postoperative computed tomography (CT) data does not match one-to-one. Thus, the displacement of each point cannot be defined. Therefore, training data cannot be generated. To solve this problem, we segment the point cloud by considering osteotomized segments and matching the segmented parts to each part after surgery. The iterative closest point (ICP) method was used to match the preoperative point cloud to the postoperative point cloud. After matching, six transformation parameters were calculated and used as labels for the training data. Through the proposed training data generation method, it is possible to use actual pre- and postoperative point cloud data during training.

The second issue relates to the architecture of the DNN. The present method divides the skull into six parts and predicts the 3D deformation parameters for each. To do this, it is necessary to define a network to learn the transformation parameters of each part. Orthognathic surgery was performed considering the overall shape of the skull. Therefore, when only the divided part is used as input, the features of the transformation to be learned cannot appear; therefore, the reference point cloud should be input. In this study, features were extracted by inputting the entire point cloud of the preoperative skull and the divided part into the feature extractor. Each extracted feature is then merged through concatenation, and, finally, the deformation parameters of each part are predicted through a fully connected layer. Additionally, the accuracies according to the feature extractors for irregular point cloud data proposed in the previous studies, such as pointnet [25], pointnet++ [26], and pointconv [27], were compared to construct a network with higher accuracy.

The third issue is related to the loss function for higher accuracy. When using the network described in the second issue, two types of point cloud data are input, and six transformation parameters are predicted. The purpose of the network is to accurately predict the six transformation parameters. Therefore, the mean absolute error (MAE) of the six transformation parameters between prediction and ground truth should be minimized. However, when learning with only the MAE of the transformation parameters, the information given to the DNN is limited, and the learning efficiency tends to decrease. To solve this issue, we defined a 3D functional transformation layer that uses divided point cloud data and predicted transformation parameters. By using the transformation layer, the predicted transformed point cloud coordinates are output, and the MAE can be calculated by comparing them to the ground truth coordinates. Finally, the loss function is defined by using the sum of the MAE of the transformation parameters and coordinates, and it is confirmed that the accuracy can be improved.

Therefore, the contributions of this study can be described as follows:

1. A new postoperative prediction method using the point coordinate information of real 3D CT data for orthognathic surgery is proposed.
2. A new training data generation method for training postoperative predictive networks is proposed.
3. A new architecture and loss function for DNNs of higher accuracy are provided.
4. In the case of the previous method, it was not possible to train using real pre- and postoperative data, but the method proposed in this study enables training using real pre- and postoperative data.

The remainder of this paper is organized as follows. First, the method for generating training data, alignment network, and loss function is described in detail. Thereafter, the results obtained using the proposed method are described. Accuracies based on the feature extractor and loss function are discussed. Finally, we summarize and discuss our findings.

## 2. Materials and Methods

### 2.1. Raw Data Acquisition

In this study, 269 participants at Wonkwang University, Daejeon Dental Hospital, were included. All were native Koreans. The patients’ chief complaints were retrognathism, prognathism, and asymmetry. Accordingly, CT images were taken for analysis before and one week after surgery. The inclusion criteria were as follows: age between 18 and 29 years, complete dentition apart from third molars, and the patient’s agreement with 3D CT of the head. Apart from cases with congenital deformities, such as cleft lip, cleft palate, and hemifacial microsomia, only patients with developmental dysmorphosis were included. All patients underwent preoperative CT after completion of preoperative orthodontic treatment. There was no case of teeth extraction for orthodontic treatment. A single surgeon diagnosed all cases, and two operated on all cases in the same way. LeFort I osteotomy was performed on the maxilla, vertical ramus osteotomy, or sagittal split ramus osteotomy of the mandible, and genioplasty on the chin. This study was approved by the Institutional Review Board of Wonkwang University Daejeon Dental Hospital (W2109/002-001). 

A SOMATOM Definition Dual Source CT (DSCT; Siemens, Forchhelm, Germany) was used to conduct a 3D analysis under the following imaging conditions: scanning time, 1 s; 100 kV; field of view, 20.8 cm; 76 mAs; and 0.5 mm thickness. For accurate evaluation, individuals were asked to maintain a centric occlusion and remain still. All CT cross-sections were saved in the Digital Imaging Communications in Medicine format.

### 2.2. Ground Truth Rigid Transformation Parameter Calculation

To train the alignment network, the ground truth values of the six rigid transformation parameters are needed. In this study, the ICP used for point cloud alignment was used to obtain this value. ICP finds a rigid body transformation where the source points best match the reference, while the reference points are fixed. The ICP finds a rigid body transformation by iteratively minimizing the error metric, expressed as the distance between the reference point cloud and the source point cloud. ICP was first proposed by Chen and Medioni and Besl and McKay, and various ICP variants have been proposed to improve the performance.

The ICP is implemented in an open-source environment, such as Meshlab, CloudCompare, or Point Cloud Library. In this study, alignment was performed using CloudCompare 2.11.3 (Anoia). As the actual preoperative and postoperative skulls are not aligned with each other, and the coordinates of the minimum point are all different. To solve this problem, in step 1, the pre- and postoperative skull were first translated so that the minimum values of each coordinate were zero. Subsequently, the entire preoperative skull was aligned based on the cranium (part 1) of the postoperative skull. In step 2, the rigid transformation parameters were calculated by aligning preoperative parts 2–4 with the corresponding postoperative parts. In this study, the RMS difference and final overlap values were set to 1 × 10^−20^ and 100%, respectively, for the convergence criteria. Using the process described above, transformation parameters for pre- and postoperative data of a total of 269 patients were calculated and used as ground truth values.

### 2.3. Data Normalization

To train the proposed network, the data were normalized. After calculating the rigid transformation parameters for all patients, the maximum value, ${\bar{y}}_{\max}$, and minimum value, ${\bar{y}}_{\min}$, of each parameter were obtained. With this value, the label value was normalized, as shown in (1):

(1) $${\bar{y}}_{i}\leftarrow \frac{{\bar{y}}_{i}-{\bar{y}}_{\min}}{{\bar{y}}_{\max}-{\bar{y}}_{\min}},\;i=1,\cdots ,\;269$$

The point cloud coordinates of all patients were also normalized, as shown in (2), using the maximum value, $p_{\max}$, among all patients and used as input.

(2) $$p_{i}\leftarrow \frac{p_{i}}{p_{\max}},\;i=1,\cdots ,\;269$$

Note that before being input into the transformation layer of the proposed network, the transformation parameter values and input coordinate values were unnormalized and used.

### 2.4. Input Point Generation for Segmentation and Alignment Network

The number of point clouds in the actual patient data was different for each patient. In this study, a certain number of points were input into the segmentation and alignment networks. In the case of segmentation, all point cloud data of patients were divided into 2048 points in order, and input data were generated by setting the batch size to 64. In the case of the alignment network, three networks were trained for parts 2–4. In the case of part 1 used as a reference, 16,384 points were input for all alignment networks, and 8192, 16,384, and 2048 points were input for parts 2, 3, and 4, respectively. For the alignment network, the input points were randomly selected from each part.

### 2.5. Three-Dimensional Postoperative Prediction Method

**Figure 1:**
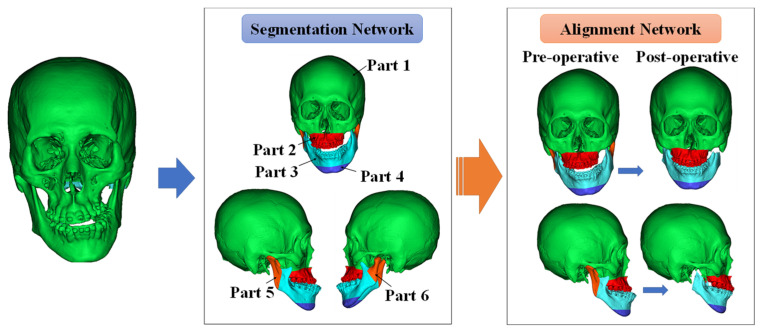
Two main stages for three-dimensional orthognathic surgery prediction.

The proposed DNN is divided into two main stages, as shown in Figure 1. The role of the first network is to segment the patient’s skull by learning the actual osteotomy area. The divided parts in the first network were used as inputs for the second network. The role of the second network is to learn the actual patient’s surgical results and align each part. The alignment network is the core method proposed in this study, and it serves to align each part based on the cranium, which does not change after surgery. Subsequently, the loss function for training the proposed network is explained.

### 2.6. Point Segmentation Network for the Jaw Part Division

Orthognathic surgery involves cutting the jaw and aligning the osteotomized segments. Therefore, the DNN also predicts surgical outcomes in a procedure, such as that of actual surgery. This subsection describes the first process: the jaw segmentation network. The jaw point cloud is segmented using a segmentation network to create each part used as an input to the alignment network. The osteotomized segments of the patient considered in this study were slightly different, depending on the condition of the patient’s jaw. To divide each part through deep learning, the osteotomized area must be generalized so that all patient data were checked. Therefore, the surgical method was generalized. As shown in Figure 1, the network was configured to divide the skulls of all patients into six parts.

There is a large difference in the number of points, depending on the segmented skull part. For the cranium (Figure 1, part 1), which occupies the largest part, there were cases in which the number of points was almost 100 times larger than that of parts 5 and 6, which had the smallest number of points. To increase the accuracy of the DNN, it is important to reduce the data imbalance among parts. Therefore, in this study, the skull was divided sequentially by dividing the cranium (part 1) and the other lower parts. After the first segmentation, the lower parts were separated into five parts using the second segmentation network. 

There are many networks for point cloud segmentation. In this study, pointnet and pointconv are used as the first segmentation network and second segmentation network, respectively. These networks are a DNN that uses point data composed of x (transverse), y (anterior–posterior), and z (vertical) coordinates in an irregular format rather than a regular one, such as an image. Pointnet is a network designed to satisfy the permutation invariant, in which the result, according to the input order of points, does not change. The rigid motion invariant that the result according to the rigid body transformation of the point also does not change. Pointconv is a density re-weighted convolution based on pointnet++ that improved pointnet. It is able to fully approximate the 3D continuous convolution on any set of 3D points.

### 2.7. The 3D Point Alignment Network for Jaw Alignment Prediction

The points segmented through the segmentation network are used as inputs to the alignment network, which is the core method of this study. The alignment network presented in this study learns 3D rigid-body transformation parameters. The 3D points can be rigidly transformed by methods, such as a transformation matrix, Euler angle, and axis and angle. In the case of the Euler angle and axis and angle methods, the transformation of a rigid body can be expressed using a total of six parameters: three related to rotation and three related to translation. In the case of the Euler angle method, the parameter values vary according to the order of the rotation axes; therefore, in this study, the network was designed to learn the parameters expressed using the axis and angle method. 

**Figure 2:**
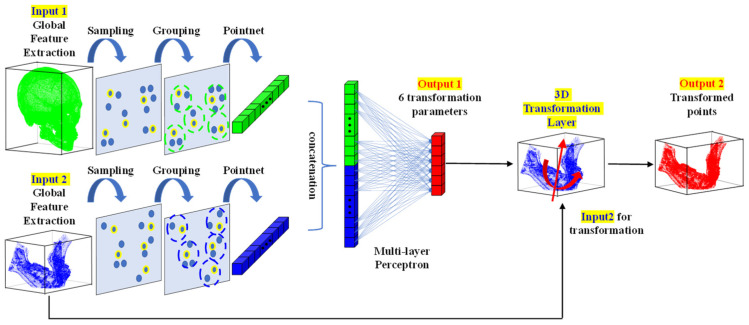
Three-dimensional point alignment network for prediction of postoperative result of orthognathic surgery.

The structure of the point alignment network is shown in Figure 2. For the global feature extraction, pointnet, pointnet++, pointconv, and others can be used. In the proposed network, the points of a whole part and a part requiring alignment are used as the reference input and aligned input, respectively. Subsequently, a feature extraction was performed for each part. The features of each input were then combined through concatenation and input into a multilayer perceptron (MLP). In the MLP, six rigid body transformation parameter values were calculated as the output, as shown in Figure 2. Additionally, the six rigid body transformation parameter values were input into the 3D functional transformation layer alongside the points of the part that needed alignment, so that the rigid-body transformed points were also calculated. In the 3D transformation layer, the final transformed point, $p^{\prime }$, was calculated by adding $p_{t}$ representing the x, y, and z translation values to the rotated point, as shown in (3):

(3) $$p^{\prime }=p\cos\theta +\left(a\times p\right)\sin\theta +a\left(a\cdot p\right)\left(1-\cos\theta \right)+p_{t}$$

### 2.8. Loss Function for Training of Point Alignment Network

The point alignment network described in the previous section learns the 3D transformation parameter values of the part through deep regression. Therefore, the MAE is used as the first loss function to match the transformation parameter value with the ground truth value as much as possible. Additionally, the mean value of distance error (DE) between points of a part is set as the second loss so that the coordinates transformed using the predicted transformation parameter value match the coordinates transformed through the ground truth value. By adding two loss functions, the final loss function is defined as shown in (4):

(4) $$Loss=\alpha \sum_{i=1}^{6}\left|y_{i}-{\bar{y}}_{i}\right|+\sum_{j=1}^{p_{n}}\left|p_{j}^{\prime }-{\bar{p}}_{j}\right|$$

where ${\bar{y}}_{i}$, $p_{j}^{\prime }$, and ${\bar{p}}_{j}$ are the ground truth values of the six rigid transformation parameters, predicted transformed points, and ground truth transformed points, respectively. Moreover, α and $p_{n}$ are the weights for the first loss and the number of points to be transformed, respectively. Each loss value is complementary to the other. If the first loss value is minimized and matches the ground truth value, the second loss value becomes zero. However, in the training process dealing with real data, the loss value rarely becomes zero. In the case of the first loss function, it is possible to have the same loss value, even if the values of each transformation parameter differ. Therefore, the second loss function was defined to train the coordinates between points to be smaller, even if the value of the first loss function was the same. In this study, the value of α was set to 10.

## 3. Results

To validate the accuracy of the proposed procedures, the training and testing data were apportioned randomly by 0.8 and 0.2, respectively. For training, Adam was used as the optimizer. The learning rate was fixed at 0.001 for training the segmentation and alignment networks, and the number of epochs for training was set to 500. The segmentation network and alignment network are trained separately and validated by using test data. The performance measures of the segmentation network and alignment network are described in the following subsections. 

### 3.1. Performance of Segmentation Network

The accuracy of the segmentation network was measured using an accuracy, mean intersection over union (mIoU) and dice coefficient (DC). As shown in the Table 1, segmentation network 1 using pointnet and segmentation network 2 using pointconv had the highest accuracy among the three segmentation networks, respectively. In the case of segmentation network 1, which divided the upper and lower parts, the highest accuracy, mIoU and DC were 0.9998, 0.9994, and 0.9998, respectively; the confusion matrix is expressed in Table 2. In the case of segmentation network 2, which divides the lower part into five segments, the highest accuracy, mIoU and DC were 0.9949, 0.9900, 0.9949, respectively; the confusion matrix is presented in Table 3. The misclassified points were mainly points between adjacent parts that were acceptable for postoperative skeletal prediction.

### 3.2. Performance of Alignment Network

The performance of the alignment network was measured using two metrics. The first is the accuracy of the transform parameter, which was calculated using (5) for each patient as the absolute error between the true label value and the prediction value.

(5) $$\left|y_{i}-{\bar{y}}_{i}\right|,\;i=1,\cdots ,6$$

The second metric is the average value of the absolute error of the transformed point coordinates between the prediction and the true labels. The metric was calculated for each patient as follows, using (6):

(6) $$\sum_{j=1}^{p_{n}}\left|p_{j}^{\prime }-{\bar{p}}_{j}\right|/p_{n}$$

**Figure 3:**
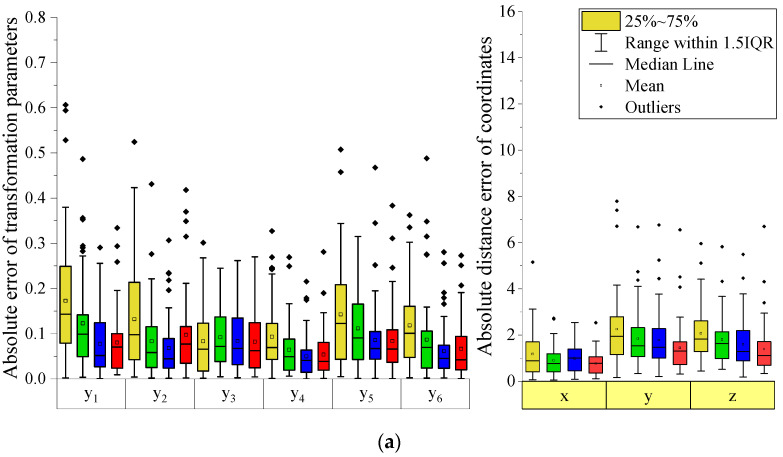

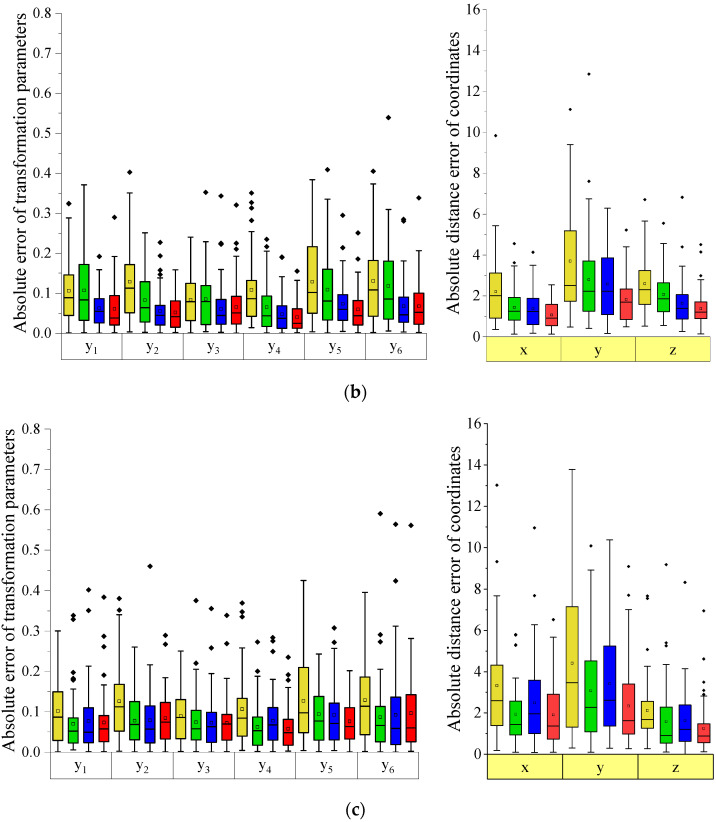
Box plot for the absolute difference of 6 transformation parameters, and coordinates for (**a**) part 2, (**b**) part 3, and (**c**) part 4. Yellow, green, blue, and red colors of the graph are error of network using pointnet, pointconv, pointnet++, and present method.

**Figure 4:**
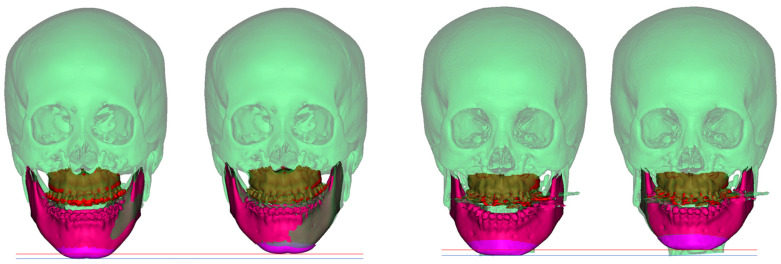

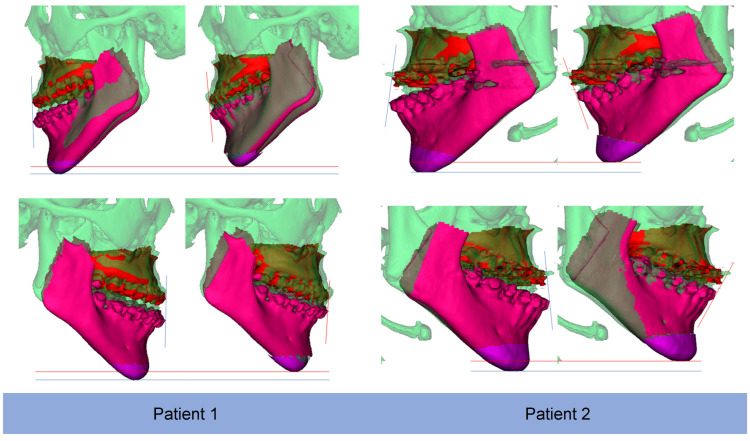
Pre-operative (**left**) and post-operative (**right**) surgery prediction through the alignment network of two randomly selected patients.

The first metric was calculated for each patient belonging to the test data, and the box plot is shown in left side of Figure 3, where (a), (b), and (c) show the absolute error of the transformation parameters of parts 2, 3, and 4, respectively. The second metric was also calculated for each patient belonging to the validation data, and a box plot for each part is shown in right side of Figure 3. As shown in the graph, the alignment network using pointnet++ as a feature extractor had the highest accuracy among the three feature extractors. When the distance error loss was used, the error of the transformation parameter tended to increase slightly; however, the distance error tended to decrease. The average error of the six transformation parameters and the average error of the transformation coordinates using pointnet++ as a feature extractor and distance error loss are listed in Table 4 and Table 5, respectively. Moreover, to check the issue related with overfitting, we divided the training data and test data in a ratio of 5:5 and performed an experiment on the alignment network of part 2. As a result, it was confirmed that overfitting did not occur, showing a similar level of accuracy as when the data were split by 8:2. Additionally, the preoperative and predicted postoperative skulls are shown in Figure 4 by randomly selecting two patients. The left and right figures represent the preoperative and predicted postoperative skulls, respectively. The true transformed postoperative skull is expressed transparently in green. In Figure 4, parts 2, 3, and 4 moved similarly to the actual postoperative results. This experiment confirmed that the proposed method is effective in predicting the postoperative results of orthognathic surgery.

## 4. Discussion

Deep learning studies are also being conducted for the diagnosis and planning of dentofacial dysmorphosis [21,22,23,24]. However, most adhere to the classic method using points, planes, and angles [21,22,23]. Among these, Xiao et al. estimated reference bony-shape models for orthognathic surgical planning using 3D point-cloud deep learning [24]. In contrast to the previous study predicting the displacement of a point using artificially generated training data, the present method predicted the transformation matrix from actual surgery data. Because of this difference, the method of training data generation was completely different, and actual pre- and postoperative surgery data were considered. To predict postoperative surgery results, the present method has two main networks: the segmentation network that divides the skull into six main parts, and the alignment network for predicting the transformation parameters of each. The efficacy of the present method was confirmed by applying it to actual patients’ surgery data.

As a result of this study, the mean absolute error of transformed points showed a value smaller than 2.34 mm in x (transverse), y (anterior–posterior), and z (vertical) (Table 5). It was suggested that accurate prediction is possible in maxilla (part 2), mandible (part 3), and chin (part 4). Figure 4 shows the case of randomly selecting two patients. In each, the left represents preoperative and the right represents postoperative. The actual patient’s condition was expressed in transparent green. Then, colors were assigned to parts 2 (red), 3 (red violet), and 4 (purple), respectively. As a result, as shown in Figure 4, it was confirmed that parts 2, 3, and 4 moved very similarly to the actual post-operative. 

For this study, we used only postoperative CT data from two surgeons. In addition, all surgeries were performed through the same diagnostic and surgical methods. As is well known, the preferred diagnosis and surgical method differs, depending on the surgeon. Therefore, if the data of several surgeons were collected, the number of samples could be increased, but it would have been more difficult for DNN to accurately predict the surgical outcome. First of all, this study confirmed that DNN can accurately predict 3D postoperative results. We think that if we can collect data from several surgeons and conduct research in the future, we will be able to get better results.

Despite hard tissue changes being important in orthognathic surgery, especially for surgery planning, soft tissues are equally important to assess the final results of the surgery. The model in this study only predicted hard tissue changes, not soft tissue changes. On the other hand, recently Ma et al. introduced a learning-based framework to speed up the simulation of postoperative facial appearances [28]. Specifically, they introduced a facial shape change prediction network (FSC-Net) to learn the nonlinear mapping from bony shape changes to facial shape changes. Combining the study of Ma et al. with ours of this study, it is expected to create a model that can be easily used in actual clinical practice.

## 5. Conclusions

Even for an experienced oral and maxillofacial surgeon, it is almost impossible to intuitively predict the postoperative results of orthognathic surgery. Therefore, we have thus far relied entirely on classical analysis based on points, planes, and angles. This is time consuming. In addition, it is also greatly influenced by the skill level of the practitioner. Even studies using 3D DNN were mostly focused on points, planes, and angles. We found in this study that DNN can predict postoperative results of orthognathic surgery without relying on reference points, planes, and angles. In addition, the results of this study were accurate enough for immediate clinical application. From this study, postoperative results can now be easily predicted by simply entering the point cloud data of CT. 

## Figures and Tables

**Table 1:** Performance evaluation for segmentation network.

	SEGMENTATION NETWORK 1			SEGMENTATION NETWORK 2		
	Pointnet	Pointnet++	Pointconv	Pointnet	Pointnet++	Pointconv
**Accuracy**	0.9998	0.9904	0.9981	0.9787	0.9842	0.9949
**mIoU**	0.9994	0.9668	0.9933	0.9532	0.9699	0.9900
**DC**	0.9998	0.9904	0.9981	0.9787	0.9842	0.9949

mIoU: mean intersection over union. DC: dice coefficient.

**Table 2:** Confusion matrix of segmentation network 1 dividing upper and lower part of skull using pointnet.

		PREDICTION	
		Upper Part	Lower Part
**Ground Truth**	Upper part	54,694,819	7003
	Lower part	3602	10,912,496

**Table 3:** Confusion matrix of segmentation network 2 dividing lower part of skull using pointconv.

		PREDICTION				
		Part 2	Part 3	Part 4	Part 5	Part 6
**Ground Truth**	Part 2	2,902,629	13,040	16	131	0
	Part 3	17,098	5,769,107	5962	0	697
	Part 4	0	15,552	854,748	26	0
	Part 5	482	2185	7	671,104	12
	Part 6	0	24	5	0	597,479

**Table 4:** Mean absolute error of transformation parameters using pointnet++ as feature extractor and distance error loss for training. Note that y_1_–y_6_ are the six rigid transformation parameters defined by axis and angle.

	y_1_	y_2_	y_3_	y_4_	y_5_	y_6_
**part 2**	0.0803	0.0832	0.0822	0.0538	0.0838	0.0666
**part 3**	0.0609	0.0515	0.0656	0.0399	0.0595	0.0675
**part 4**	0.0733	0.0843	0.0722	0.0580	0.0760	0.0964

**Table 5:** Mean absolute error of transformed points using pointnet++ as a feature extractor and distance error loss for training where x, y, and z are transverse, anterior–posterior, and vertical distance error, respectively.

	x (mm)	y (mm)	z (mm)
**part 2**	0.765	1.455	1.392
**part 3**	1.069	1.831	1.375
**part 4**	1.913	2.340	1.257

## Data Availability

The datasets generated and/or analyzed during the current study are available from the corresponding author upon reasonable request, subject to the permission of the institutional review boards of the participating institutions.
